# Theoretical foundations of the sound analog membrane potential that underlies coincidence detection in the barn owl

**DOI:** 10.3389/fncom.2013.00151

**Published:** 2013-11-08

**Authors:** Go Ashida, Kazuo Funabiki, Catherine E. Carr

**Affiliations:** ^1^Department of Biology, University of MarylandCollege Park, MD, USA; ^2^Systems Biology, Osaka Bioscience InstituteSuita, Osaka, Japan; ^3^Division of Biology, California Institute of TechnologyPasadena, CA, USA

**Keywords:** phase-locking, sound localization, auditory brainstem, periodic signals, oscillation, owl

## Abstract

A wide variety of neurons encode temporal information via phase-locked spikes. In the avian auditory brainstem, neurons in the cochlear nucleus magnocellularis (NM) send phase-locked synaptic inputs to coincidence detector neurons in the nucleus laminaris (NL) that mediate sound localization. Previous modeling studies suggested that converging phase-locked synaptic inputs may give rise to a periodic oscillation in the membrane potential of their target neuron. Recent physiological recordings *in vivo* revealed that owl NL neurons changed their spike rates almost linearly with the amplitude of this oscillatory potential. The oscillatory potential was termed the sound analog potential, because of its resemblance to the waveform of the stimulus tone. The amplitude of the sound analog potential recorded in NL varied systematically with the interaural time difference (ITD), which is one of the most important cues for sound localization. In order to investigate the mechanisms underlying ITD computation in the NM-NL circuit, we provide detailed theoretical descriptions of how phase-locked inputs form oscillating membrane potentials. We derive analytical expressions that relate presynaptic, synaptic, and postsynaptic factors to the signal and noise components of the oscillation in both the synaptic conductance and the membrane potential. Numerical simulations demonstrate the validity of the theoretical formulations for the entire frequency ranges tested (1–8 kHz) and potential effects of higher harmonics on NL neurons with low best frequencies (<2 kHz).

## Introduction

Synchronized neural activity underlies various types of information processing in the brain. A diversity of sensory neurons encode temporal information via phase-locked spiking (Carr and Friedman, 1999). Phase-locking, or the generation of action potentials at a certain phase of the reference signal, is prevalent in the auditory system (Oertel, 1999; Ashida et al., 2010; Brette, 2012). In the auditory brainstems of mammals, reptiles, and birds, neurons involved in sound localization convey precise temporal information of sound using phase-locked spikes (cats: Joris et al., 1994; gerbils: Dehmel et al., 2010; caimans: Carr et al., 2009; owls: Sullivan and Konishi, 1984; Köppl, 1997; chickens: Warchol and Dallos, 1990; Fukui et al., 2006; redwing blackbirds: Sachs and Sinnott, 1978). Among various animal species tested, auditory neurons in the barn owl show the highest temporal acuity with a precision of less than 0.1 ms (Köppl, 1997). The degree of phase-locking, measured as the vector strength (VS) (Goldberg and Brown, 1969), is significant for frequencies up to about 8 kHz in the owl's nucleus magnocellularis (NM) (Sullivan and Konishi, 1984; Köppl, 1997).

**Figure 1:**
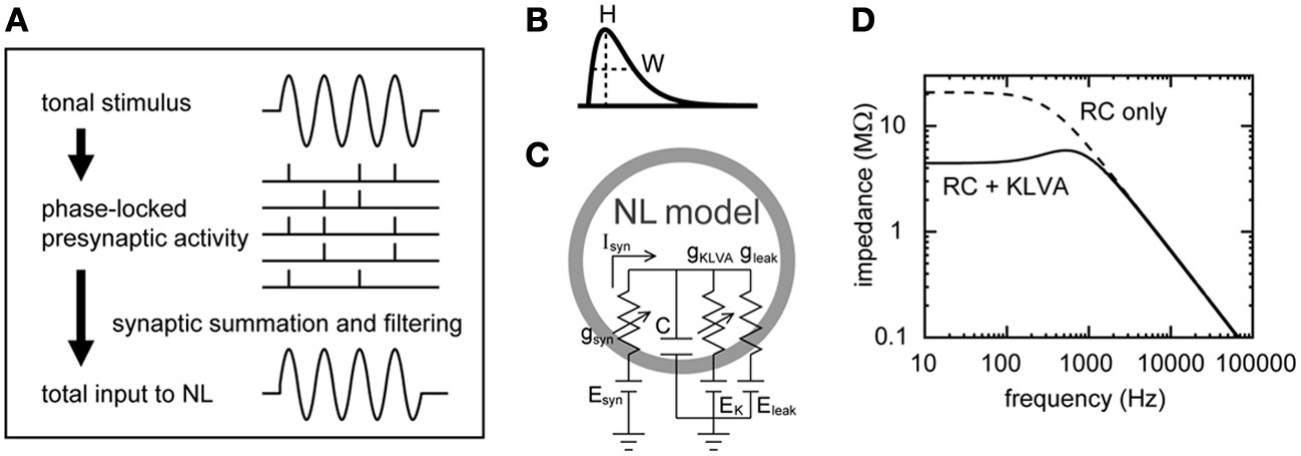
**Schematic drawings of the synaptic input and the membrane response of the NL neuron model. (A)** Formation of the oscillatory synaptic input. Tonal stimuli induce phase-locked spiking in NM fibers that converge on an NL neuron, creating a periodic oscillation in the synaptic input to NL. For clarity, higher harmonics and noise components are not included in this schematic drawing. **(B)** Alpha-function as the model unitary synaptic input. The half peak width *W* determines the speed of rise and decay, while *H* is the peak height of the curve (see text for equations). **(C)** Single compartment NL neuron model (Funabiki et al., 2011). Leak and low-voltage-activated potassium (*K*_LVA_) conductances are included in the membrane. **(D)** Linear membrane impedance of the model neuron. Introduction of the *K*_LVA_ conductance greatly reduces membrane impedance below 1–2 kHz.

Both mammals and birds have specialized neural circuits to compute the interaural time difference (ITD), which is one of the most important cues for sound localization (see Joris and Yin, 2007; Grothe et al., 2010; Ashida and Carr, 2011, for reviews). In the avian brainstem, axons from the NM form delay lines and provide phase-locked spike outputs while their target neurons in the nucleus laminaris (NL) detect coincident synaptic inputs and change their spike rates with ITD (Carr and Konishi, 1990; Köppl and Carr, 2008). Previous modeling results suggested that a convergence of phase-locked spikes creates an oscillatory synaptic input whose period is the same as that of the stimulus tone (Figure 1A; Gerstner et al., 1996; Reyes et al., 1996; Kempter et al., 1998; Ashida et al., 2007; Slee et al., 2010). Recent *in vivo* intracellular recordings revealed that the barn owl's NL neurons indeed show oscillating membrane potentials (Funabiki et al., 2011). This oscillation was termed the “sound analogue potential” because its waveform resembled the waveform of the stimulus tone delivered to the owl's ears. Both physiological (Funabiki et al., 2011) and modeling (Ashida et al., 2007) results showed that the amplitude of the sound analog potential changes periodically with ITD, and that the NL neurons vary their spike rates almost linearly to this oscillation amplitude. In the following text, the main oscillatory component is therefore referred to as the “signal” or “AC,” whereas the average input level is called the “DC.” The DC component was shown to be irrelevant to the ITD computation in NL (Funabiki et al., 2011). All the frequency components other than the AC and DC are regarded as “noise” because they do not encode ITDs (see Reyes et al., 1996; Ashida et al., 2007; Slee et al., 2010 for related discussion).

Our previous simulations demonstrated that, if appropriate parameters are chosen, sound analog potentials can be quantitatively reproduced by the NM-NL model (Ashida et al., 2007; Funabiki et al., 2011). In this model, phase-locked spikes of NM fibers (Figure 1A) are described by an inhomogeneous Poisson process (Gerstner et al., 1996; Kempter et al., 1998; Shimokawa et al., 1999; Burkitt and Clark, 2001; Kuhlmann et al., 2002; Grau-Serrat et al., 2003); unitary synaptic inputs (Figure 1B) are modeled by an alpha-function (Gerstner and Kistler, 2003); and the responses of the NM membrane are simulated by a conductance-based single-compartment model (Figures 1C,D) (Ashida et al., 2007; Funabiki et al., 2011) with leak and low threshold potassium conductances (*K*_LVA_), which has been shown to benefit fine temporal coding (e.g., Svirskis et al., 2002; Gai et al., 2009; Jercog et al., 2010; Mathews et al., 2010). In this paper, we analyze the model in detail and theoretically formulate how phase-locked NM inputs lead to the sound analog potentials in NL. The primary goals of this paper are two-fold: (1) to relate the model parameters to the DC, AC, and noise components of the synaptic input and membrane potential combining the Poisson process with linear membrane impedance analysis techniques (e.g., Hutcheon and Yarom, 2000); (2) to test the validity of the theoretical descriptions using numerical simulation of the NM-NL model. In the accompanying paper (Ashida et al., 2013), we apply our theoretical results obtained in the present paper to investigate how presynaptic, synaptic, and postsynaptic factors may affect ITD coding in the NL neuron.

## Materials and methods

### Phase-locked spiking of presynaptic fibers

Following previous studies, we use the inhomogeneous Poisson process to model phase-locked spiking activity (Gerstner et al., 1996; Kempter et al., 1998; Shimokawa et al., 1999; Burkitt and Clark, 2001; Kuhlmann et al., 2002; Grau-Serrat et al., 2003; Ashida et al., 2007; Kuokkanen et al., 2010). Output spikes of each NM neuron are modeled as an inhomogeneous Poisson sequence *n*(*t*) with a periodic intensity function λ (*t*) = λ_0_(1 + ∑^∞^_*k* = 1_ *a_k_* cos(2π*k*ν*t* + η_*k*_)), where λ_0_ is the mean intensity, *a_k_* (*k* = 1, 2, …) is the strength of the *k*-th frequency component, ν is the fundamental frequency (i.e., 1/ν is the period), η_*k*_ is the phase of the *k*-th component. The spike train *n*(*t*) is regarded as a sum of delta functions: *n*(*t*) = ∑^*N*^_*j* = 1_ δ(*t − t_j_*), where *N* is the total number of spikes in the sequence, and *t_j_* is the timing of the *j*-th spike. The degree of phase-locking of a spike sequence is measured as the vector strength *r* (Goldberg and Brown, 1969), which is defined as $r=\frac{1}{N}\sqrt{{\left(\sum_{j=1}^{N}\cos(2\pi f\;t_{j})\right)}^{2}+{\left(\sum_{j=1}^{N}\sin(2\pi f\;t_{j})\right)}^{2}}$, with *f* being the reference frequency. In the following text, we assumed that *f* = ν (i.e., we focus on the locking to the fundamental frequency) unless otherwise mentioned. For the inhomogeneous Poisson sequence introduced above, the *VS* is related to the intensity function as *r* = *a*_1_/2.

The power spectral density (PSD) *P^n^*(*f*) of the sequence *n*(*t*) can be calculated as

(1) $$P^{n}(f)=\lambda_{0}+\lambda_{0}^{2}\text{​}\left(\text{​}\delta (f)+\sum_{k=1}^{\infty }\text{​}\frac{a_{k}^{2}}{4}\left(\delta (f-k\nu )+\delta (f+k\nu )\right)\right)\text{​},$$

with δ(*f*) being the delta function (Wiesenfeld et al., 1994; Hohn and Burkitt, 2001; Kuokkanen et al., 2010). The first term λ_0_ corresponds to the noise or the randomness of the sequence, the second term λ^2^_0_δ(*f*) corresponds to the mean strength of the sequence, and the remaining term corresponds to the fundamental frequency component and higher order harmonics. If the sequence is not infinitely long but has a time length *T*, the PSD becomes

(2) $$P_{T}^{n}(f)=\lambda_{0}+\lambda_{0}^{2}\left(T\delta_{f}+\sum_{k=1}^{\infty }\frac{a_{k}^{2}T}{4}\left(\delta_{f-k\nu }+\delta_{f+k\nu }\right)\right),$$

where δ_*f*_ = 1 (for *f* = 0) and δ_*f*_ = 0 (otherwise).

### Circular distributions

For the periodic intensity function λ(*t*), the von Mises distribution and the wrapped Gaussian distribution have been most widely used (Fisher, 1993; Gerstner et al., 1996; Kempter et al., 1998; Kuhlmann et al., 2002; Grau-Serrat et al., 2003; Ashida et al., 2007).

**Figure 2:**
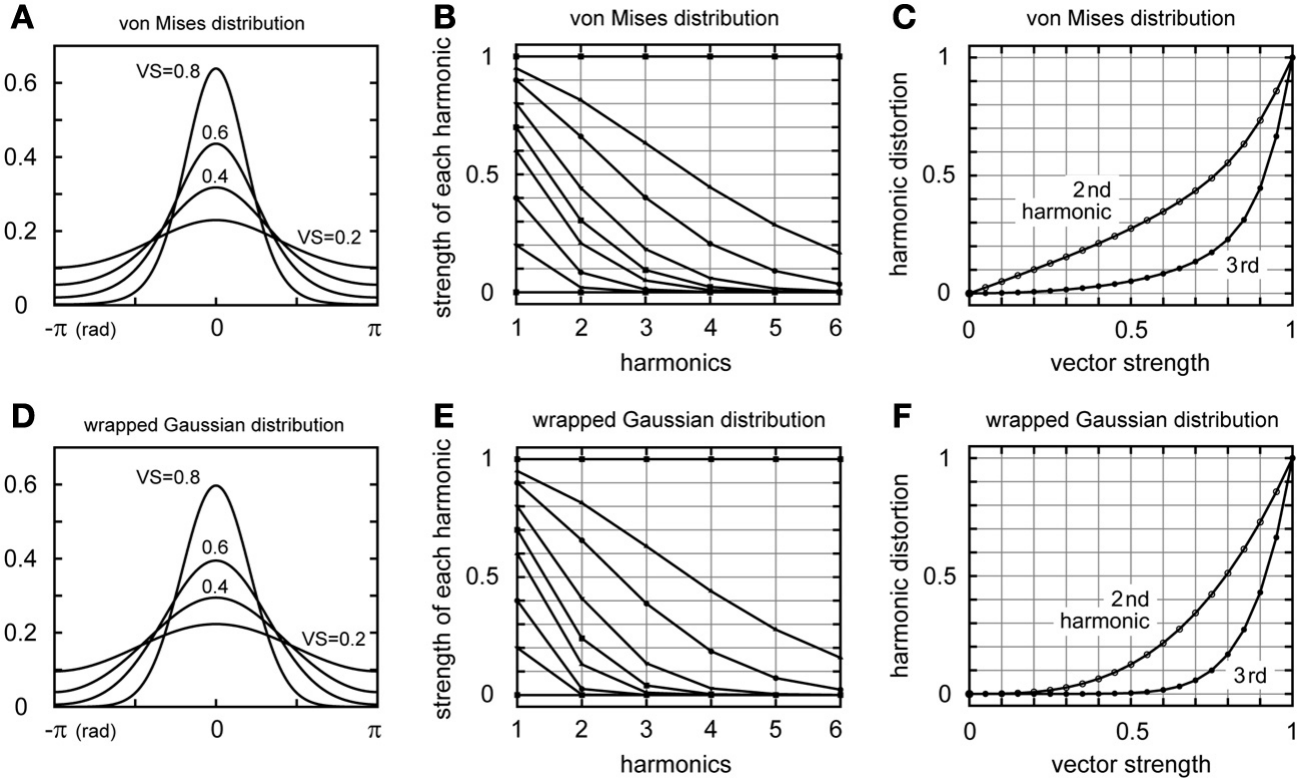
**Periodic distributions and higher harmonics. (A)** The von Mises distribution. Curves with *VS* = 0.2, 0.4, 0.6, and 0.8 are shown (see below for the values of the concentration parameter κ). **(B)** Strengths of the first harmonic (fundamental frequency) and higher harmonics of the von Mises distribution. Each curve shows the strength of the first harmonic (*VS*) and corresponding higher harmonics. **(C)** Second and third harmonic distortion of the von Mises distribution. **(D)** The wrapped Gaussian distribution. Curves with *VS* = 0.2, 0.4, 0.6, and 0.8 are shown (see below for the values of the dispersion σ. **(E)** Strengths of the first harmonic (fundamental frequency) and higher harmonics of the wrapped Gaussian distribution. Each curve shows the strength of the first harmonic (*VS*) and corresponding higher harmonics. **(F)** Second and third harmonic distortion of the wrapped Gaussian distribution. In **(B,E)**, nine curves (*VS* = 0, κ = 0, σ = ∞; *VS* = 0.2, κ = 0.408, σ = 1.794; *VS* = 0.4, κ = 0.874, σ = 1.353; *VS* = 0.6, κ = 1.516, σ = 1.011; *VS* = 0.7, κ = 2.014, σ = 0.845; *VS* = 0.8, κ = 2.871, σ = 0.668; *VS* = 0.9, κ = 5.305, σ = 0.459; *VS* = 0.95, κ = 10.27, σ = 0.320; *VS* = 1; κ = ∞, σ = 0) are drawn to show the decaying patterns of harmonics.

The von Mises distribution (Figure 2A) is defined as $p_{\kappa }(x)=\frac{1}{2\pi I_{0}(\kappa )}\exp(\kappa \cos(x-x_{0}))$, where κ is the concentration parameter and *x*_0_ is the initial phase. *I*_0_(κ) is the modified Bessel function of order zero assuring that ∫^π^_−π_*p*_κ_(*x*)*dx* = 1. In the following text, we set *x*_0_ = 0 for simplicity. Since *p*_κ_(*x*) is a 2π-periodic function, it can be expanded as a sum of cosine functions $p_{\kappa }(x)=\frac{r_{0}}{2\pi }+\frac{1}{\pi }\sum_{n=1}^{\infty }r_{n}\cos(nx)$. The coefficient *r_n_* can be calculated as $r_{n}=\int_{-\pi }^{\pi }p_{\kappa }(x)\cos(nx)dx=\frac{I_{n}(\kappa )}{I_{0}(\kappa )}$, where *I_n_*(κ) denotes the modified Bessel function of order *n* (Abramowitz and Stegun, 1972). Note that *r*_0_ = *I*_0_(κ)/*I*_0_(κ) = 1. The *VS* at the *n*-th harmonic is thus *r_n_*/*r*_0_ = *I_n_*(κ)/*I*_0_(κ) (Figure 2B). The harmonic distortion, defined as the ratio of the n-th harmonic to the fundamental component, is *r_n_*/*r*_1_ = *I_n_*(κ)/*I*_1_(κ) (Figure 2C).

The wrapped Gaussian distribution (Figure 2D) is defined as $g_{\sigma }(x)=\frac{1}{\sigma \sqrt{2\pi }}\sum_{k\;=\;-\;\infty }^{\infty }\exp\left(-\frac{{\left(x-2\pi k\right)}^{2}}{2\sigma^{2}}\right)$, where σ denotes the dispersion of the distribution. Note that ∫^π^_−π_*g*_σ_(*x*)*dx* = 1. Since *g*_σ_(*x*) is a 2π-periodic function, it can be expanded as a sum of cosine functions $g_{\sigma }(x)=\frac{R_{0}}{2\pi }+\frac{1}{\pi }\sum_{n=1}^{\infty }R_{n}\cos(nx)$. The coefficient *R_n_* can be calculated as $R_{n}=\int_{-\pi }^{\pi }g_{\sigma }(x)\cos(nx)dx=\exp\left(-\frac{n^{2}\sigma^{2}}{2}\right)$ (Anderson, 1973). Note that *R*_0_ = 1. The *VS* at the *n*-th harmonics is *R_n_*/*R*_0_ = exp(−*n*^2^σ^2^/2) (Figure 2E). The harmonic distortion, defined as the ratio of the *n-th* harmonic to the fundamental component, is *R_n_*/*R*_1_ = exp(−*n*^2^σ^2^/2)/ exp(−σ^2^/2) = exp(−(*n*^2^−1)σ^2^/2) (Figure 2F).

The von Mises distribution and the wrapped Gaussian distribution have, in general, very similar shaped curves (Figures 2A,D). Their higher harmonics decrease rapidly for *VS* < 0.7 (Figures 2B,E). If the *VS* is higher than 0.7, higher harmonics need to be considered in estimating the noise component (see also Discussion). Especially in the case of perfect phase-locking (*VS* = 1.0), these distributions become a delta function and all the higher harmonics have vector strengths of 1.0. The possible effects of higher harmonics will be discussed later. Fisher (1993) points out that the above two distributions are hard to distinguish in practical applications. Prior modeling studies of phase-locking used either the von Mises distribution (e.g., Grau-Serrat et al., 2003; Ashida et al., 2007) or the wrapped Gaussian distribution (e.g., Gerstner et al., 1996; Kempter et al., 1998; Kuhlmann et al., 2002). Nevertheless, comparison of these models in terms of neuronal coding will be a subject of future study. In this paper, we use the von Mises distribution for our simulation.

### Simulating phase-locked spike sequences

In our simulations, we modeled phase-locked input from each NM fiber using an inhomogeneous Poisson process with a time-dependent periodic intensity function λ(*t*) = 2πλ_0_ *p*_κ_ (2π*f_s_t*), where *f_s_* is the frequency of the stimulus tone and λ_0_ is the mean intensity (= mean spike rate). The degree of phase-locking measured by vector strength *r* can be related to the concentration parameter κ as *r* = *I*_1_(κ)/*I*_0_(κ). We assumed that all the NM fibers were mutually independent but locked to the same phase of the stimulus tone with single *VS* (Kuokkanen et al., 2010). Note that we considered only the “best ITD” situation where all the ipsi- and contralateral NM inputs arrived perfectly in-phase because ITD dependence of the phase-locked synaptic input has already been examined in our previous study (Ashida et al., 2007). The parameters used in the model are summarized in Table 1.

**Table 1:** **Parameter values used in the simulation of synaptic inputs**.

**Parameter**	**Value**
Stimulus sound frequency	1000–8000 (Hz)
Mean spiking rate of each NM fiber	500 (Hz)
Number of NM fibers converging onto one NL cell	300
Vector strength of phase-locked NM spiking	0.6
Half peak width W of unitary EPSG	0.1 (ms)
Magnitude H of unitary EPSG (alpha function)	1.3 (nS)

These values, except for the stimulus frequency, are the same as those used in our previous study (Funabiki et al., 2011) and fixed in this paper. The number and the mean spike rate of the NM fiber are taken from previous anatomical (Carr and Boudreau, 1993a) and physiological (Peña et al., 1996) studies. How each of these parameters affects the formation of the oscillatory potential and ITD coding will be examined in the accompanying paper (Ashida et al., 2013).

### Synaptic input

The excitatory postsynaptic conductance (EPSG) in the NL neuron induced by each presynaptic NM spike was modeled by an alpha function α(*t*) = (*Ht*/τ) exp(1 − *t*/τ) (*t* ≥ 0), with *H* = α(τ) being the peak height and τ being the time constant (Figure 1B). The half peak width *W* of the alpha function can be calculated by solving α(*t*) = *H*/2. The two solutions of this equation are *t*_0_ = −τ *W*_0_(−1/2*e*) and *t*_1_ = −τ *W*_−1_(−1/2*e*), where *W*_0_ is the principal real branch and *W*_−1_ is the other real branch of the Lambert *W* function (Corless et al., 1996). Therefore, the half peak width W of the alpha function is obtained as *W* = *t*_1_ − *t*_0_ = τ (*W*_0_(−1/2*e*) − *W*_ − 1_(−1/2*e*)) = 2.446τ. Note that the half peak width *W* is linear to the time constant τ (i.e., if the time constant τ is doubled, then the half peak width *W* is also doubled). The Fourier transform *F*_α_(*f*) of the alpha-function α(*t*) satisfies the equation

(3) $$\left|F_{\alpha }(f)\right|=\left|\int_{0}^{\infty }\alpha (t)\exp(-2\pi ift)dt\right|=\frac{S}{1+{\left(2\pi f\tau \right)}^{2}},$$

where *S* = *eH*τ is the area between the alpha function and the *t*-axis.

The compound synaptic input conductance *g*_syn_(*t*) is the sum of all the NM spikes filtered by the alpha function:

(4) $$g_{\text{syn}}(t)=\sum_{m=1}^{M}\sum_{i=1}^{I_{m}}\alpha (t-t_{m}^{i}),$$

where *t^i^_m_* denotes the timing of the *i*-th spike of the *m*-th NM fiber, *M* is the number of NM fibers, and *I_m_* is the number of spikes of the *m*-th fiber.

### Simulating unitary synaptic inputs

In our simulations, the values of the half peak width *W* (= 2.446τ) and height *H* (Table 1) were determined so as to reproduce the sound analogue potentials observed in experiments (Funabiki et al., 2011). With these parameter values, the average total conductance is *D_G_* = *SM*λ_0_ = *eH*τ *M*λ _0_ = 21.7 nS [see Equation (5) in Results]. Note that, since we are focusing on the steady state ITD computation in NL, transient effects such as short term synaptic plasticity (Kuba et al., 2002; Cook et al., 2003) are not explicitly included in the model; the value of *H* is assumed to be at the corresponding steady state input level.

### Simulating NL Membrane

A Hodgkin-Huxley type conductance-based single compartment model (Hodgkin and Huxley, 1952; Koch, 1999; Gerstner and Kistler, 2003) was used to simulate the membrane potential dynamics of the NL neuron (Figure 1C). The model equations and parameters (Table 2) are the same as those we used in our previous study (Funabiki et al., 2011). The single somatic compartment has leak and *K*_LVA_ conductances. The amount of these conductances were determined so that the membrane resistance of the soma at −61 mV would be about 4.4 MΩ (membrane time constant was about 0.1 ms), similar to the experimental data (Funabiki et al., 2011). The membrane capacitance was determined from the reported size and shape of the NL neuron (Carr and Konishi, 1990; Carr and Boudreau, 1993a). The slow GABAergic input (Funabiki et al., 1998; Kuo et al., 2009), which does not lock to high frequency stimuli (Yang et al., 1999; Coleman et al., 2011), and other slow conductances such as *I*_*h*_ (Yamada et al., 2005; Khurana et al., 2011), were implicitly included in the constant leak conductance. Sodium and high voltage activated potassium conductances, which are required for spike generation, were not included in the model, because spikes in the NL neuron are considered to be generated at the first node of Ranvier (Funabiki et al., 2011) located about 60 μm away from the soma (Carr and Boudreau, 1993b) and because spike generation at the node does not significantly affect the integration of the synaptic input at the soma (Ashida et al., 2007). All the synaptic input is considered to occur at the cell body because the dendrites surrounding the soma of the owl's NL are short and stubby (Carr and Konishi, 1990; Carr and Boudreau, 1993a; Kuokkanen et al., 2010). Numerical integration was performed by using the forward Euler method with a time increment of 0.1 μ s.

**Table 2:** **Equations and parameters of the model NL neuron**.

**Variable/parameter**	**Equation/value**
Membrane potential *V*(*t*)	$C\frac{d}{dt}V(t)=I_{L}+I_{\text{KLVA}}+I_{\text{syn}}$
Leak current	*I_L_* = *g_L_*(*E_L_* − *V*)
*K*_LVA_ current	*I*_KLVA_ = *ḡ_K_ d*(*V, t*)(*E_K_* − *V*)
Synaptic current	*I*_syn_ = *g*_syn_ (*E*_syn_ − *V*)
*K*_LVA_ channel activation *d*(*V, t*)	$\tau_{d}\frac{d}{dt}d(V,t)=-d(V,t)+d_{\infty }(V)$
	τ*_d_*(*V*) = *Q*^(*T* − 23)/10^_10_/[α_*d*_(*V*) + β_*d*_(*V*)]
	*d*_∞_(*V*) = α_*d*_(*V*)/[α_*d*_(*V*) + β_*d*_(*V*)]
	α_*d*_(*V*) = 0.20exp[(*V* + 60)/21.8]
	β_*d*_(*V*) = 0.17exp[−(*V* + 60)/14]
Membrane capacitance *C*	24 pF
Leak conductance *g_L_*	48 nS
*K*_LVA_ conductance *ḡ_K_*	192 nS
Reversal potential of leak current *E_L_*	−60 mV
Reversal potential of potassium current *E_K_*	−75 mV
Reversal potential of synaptic current *E*_syn_	0 mV
Temperature coefficient *Q*_10_	2.5
Temperature *T*	40°C

*The model consists of membrane capacitance, leak conductance, and K_LVA_ conductance. The kinetics of the K_LVA_ conductance was taken from a study of chicken NM (Rathouz and Trussell, 1998). Parameter values are the same as those used in our previous study (Funabiki et al., 2011) and fixed in this paper. The membrane potential V is in millivolts. The units for α_d_(V) and β_d_(V) are 1/ms*.

### Analysis of simulation data

**Figure 3:**
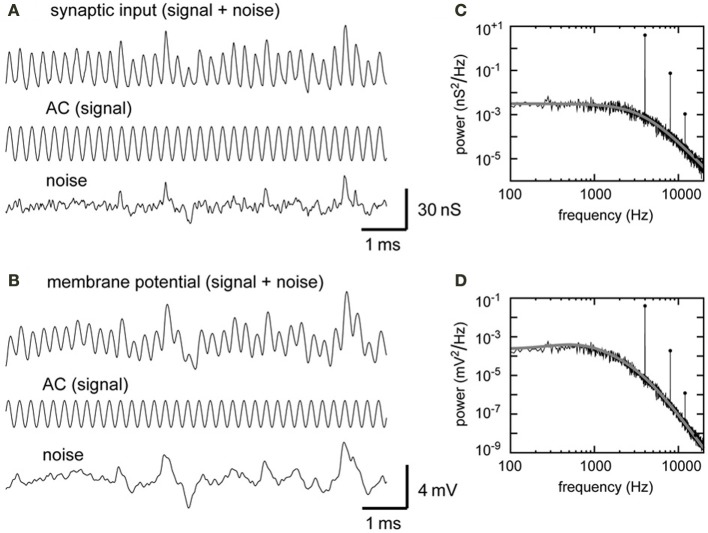
**Model synaptic input and model NL membrane potential. (A)** Simulated synaptic input conductance. The compound synaptic input, its signal component, and its noise component are shown. **(B)** Simulated membrane potential with its signal and noise components. The synaptic input shown in **(A)** (top trace) was injected to the model membrane (shown in Figure 1C). The AC components (center traces in **A,B**) were obtained from cosine fitting. The noise component (bottom traces in **A,B**) was obtained by subtracting AC (center traces in **A,B**) from the total input (top traces in **A,B**) (see Materials and Methods for detail). Input frequency = 4 kHz. **(C)** PSD of the input trace shown in **(A)**. A sharp peak appears at the stimulus frequency (4 kHz) and smaller peaks appear at higher harmonics. **(D)** PSD of the potential trace shown in **(B)**. Gray lines and filled circles in **(C,D)** show analytically predicted values.

We obtained 1100-ms-long simulated traces of conductance input and membrane potential for each parameter set. Discarding the first and the last 50 ms, we used 1000-ms traces for further analyses. To extract the component which oscillates at the stimulus frequency, a trace *x*(*t*) was fitted by a cosine function *y*(*t*) = *D*_0_ + *A*_0_ cos(2π*f_s_t* + ϕ), with *f_s_* being the stimulus frequency, *t* being time, and ϕ being the phase shift. *D*_0_ and *A*_0_ of the fitting function were, respectively, regarded as the “DC amplitude” and the “AC amplitude” of the trace. By subtracting the fitting cosine function *y*(*t*) from the original trace *x*(*t*), we obtained the “noise trace” *z*(*t*) = *x*(*t*) − *y*(*t*). The time-averaged standard deviation of the noise trace *z*(*t*) was regarded as the “noise amplitude” (see Figures 3A,B for an example).

In the frequency analyses, the 1000-ms trace was broken into ten 100-ms segments and resampled at 327,680 Hz. Each segment, consisting of 32,768 (= 2^15^) data points, was Fourier-transformed with the frequency resolution being 10 Hz and the Nyquist frequency being 160 kHz. To derive the PSD, the absolute values of the Fourier transform were squared and averaged over the 10 segments to reduce jitter in the PSD curve (Bair et al., 1994).

## Results

Phase-locked spiking activity of converging presynaptic fibers gives rise to oscillatory membrane potential to the target neuron (Gerstner et al., 1996; Reyes et al., 1996; Kempter et al., 1998; Ashida et al., 2007; Slee et al., 2010). In the following sections, we derive analytical expressions that relate the model parameters to the DC (average input), AC (signal at the locked frequency), and noise (other frequency components) levels of the model synaptic input and the membrane potential. Then we test our theoretical results using simulations.

### DC, AC, and noise of the synaptic input

We first consider the inhomogeneous Poisson spike sequence filtered by the synaptic process modeled by an alpha function (see Materials and Methods for definitions). The filtered synaptic input *x*(*t*) of each input fiber spiking at an average rate of λ_0_ can be written as the convolution of the spike sequence and the alpha function, i.e., *x*(*t*) = *n*(*t*)^*^α(*t*). The power spectrum *P^x^*(*f*) of the filtered sequence is *P^x^*(*f*) = *P^n^*(*f*)|*F*_α_(*f*)|^2^. Using the Equations (1) and (3), the integration of *P^x^*(*f*) over the entire frequency range (−∞, ∞) can be calculated as

$$\int_{-\infty }^{\infty }P^{x}(f)df=\lambda_{0}\frac{S^{2}}{4\tau }+\lambda_{0}^{2}S^{2}+\sum_{k=1}^{\infty }\frac{\lambda_{0}^{2}a_{k}^{2}}{2}{\left(\frac{S}{1+{\left(2\pi k\nu \tau \right)}^{2}}\right)}^{2}.$$

Thus the standard deviation of the noise is $\frac{S}{2}\sqrt{\frac{\lambda_{0}}{\tau }}$, the DC component of the sequence is λ_0_*S* (see Equation (1) and following text). The AC component (at the fundamental frequency ν) is $\frac{2r\lambda_{0}S}{1+{\left(2\pi \nu \tau \right)}^{2}}$, with *r* being the *VS* (note that Peak = $\sqrt{2}$RMS). If there are M independent sources of the inhomogeneous Poisson spike sequences locked to the same phase, λ_0_ is replaced by *M*λ_0_.

Therefore the average magnitude *D_G_* of the compound synaptic input conductance *g*_syn_(*t*) (Equation 4) is

(5) $$D_{G}=SM\lambda_{0},$$

where *S* = *eH*τ. The magnitude *A_G_* of the signal component of the input conductance *g*_syn_(*t*) is

(6) $$A_{G}=\frac{2SMr\lambda_{0}}{1+{\left(2\pi f_{s}\tau \right)}^{2}}=\frac{2rD_{G}}{1+{\left(2\pi f_{s}\tau \right)}^{2}},$$

with *r* being the *VS* of the input spike sequences which are phase-locked to the stimulus frequency *f_s_*. Similarly, the magnitude *L_k_* of the *k*-th harmonic is

(7) $$L_{k}=\frac{2r_{k}D_{G}}{1+{\left(2\pi kf_{s}\tau \right)}^{2}},$$

where *r_k_* is the *VS* at the *k*-th harmonic frequency (e.g., Figure 2B). Note that *A_G_* = *L*_1_. The magnitude *N_G_* of noise measured by standard deviation is

(8) $$N_{G}=\frac{S}{2}\sqrt{\frac{M\lambda_{0}}{\tau }}=\frac{D_{G}}{2\sqrt{M\lambda_{0}\tau }}.$$

Equations 5, 6, and 8 relate the DC, AC, and noise components of the synaptic input to the model parameters. Both *A_G_* and *N_G_* are linear to the average input level *D_G_*. *A_G_* is also linear to the *VS* denoted by *r* and decays with input frequency *f_s_* due to the low-pass property of the synaptic input.

### Linearized response of the single compartment NL membrane

Following Mauro et al. (1970), Koch (1999), and Richardson et al. (2003), we derive the linear membrane impedance of the RC membrane with *K*_LVA_ conductance. The dynamics of the membrane potential *V*(*t*) and the *K*_LVA_ activation variable *d*(*V, t*) are, respectively, written as:

$$\begin{array}{l} \;C\frac{d}{dt}V(t)=g_{L}(E_{L}-V)+{\bar{g}}_{K}d(V,t)(E_{K}-V)+I_{\text{ext}},\\ \tau_{d}\frac{d}{dt}d(V,t)=-d(V,t)+d_{\infty }(V), \end{array}$$

with *I*_ext_ being the external input. We linearize these equations around the holding potential *V* = V_*_. By denoting *v*(*t*):= *V*(*t*) − V_*_ and δ (*v, t*): = *d*(*V, t*) − *d*_∞_(V_*_), we have

$$\begin{array}{l} \;C\frac{dv}{dt}=-\left(g_{L}+{\bar{g}}_{K}d_{\infty }(V_{*})\right)v+{\bar{g}}_{K}(E_{K}-V_{*})\delta +\delta v+I_{0},\;\;\;\;\\ \tau_{d}\frac{d\delta }{dt}=-\delta -d_{\infty }(V_{*})+d_{\infty }(V), \end{array}$$

where *I*_0_:= *g_L_*(*E_L_* − V_*_) + *ḡ_K_d*_∞_(V_*_)(*E_K_* − V_*_) + *I*_ext_. Assuming that the displacement from the holding potential V_*_ is small, we fix τ _*d*_ at V_*_, drop the second order term δ *v*, and use the linear approximation $\frac{d_{\infty }(V)-d_{\infty }(V_{*})}{V-V_{*}}=\frac{d}{dV}d_{\infty }(V_{*})$. Now we obtain

$$\begin{array}{l} \;C\frac{dv}{dt}=-\left(g_{L}+{\bar{g}}_{K}d_{\infty }(V_{*})\right)v-{\bar{g}}_{K}(V_{*}-E_{K})\delta +I_{0},\\ \tau_{d}{}^{*}\frac{d\delta }{dt}=d_{*}^{\prime }v-\delta , \end{array}$$

where τ^*^_d_:= τ_*d*_(V_*_) and $d_{*}^{\prime }:=\frac{d}{dV}d_{\infty }(V_{*})$. Introducing a new variable *w*:= δ/*d*′_*_, and new parameters *g_v_*: = *g_L_* + *ḡ_K_d*_∞_(V_*_), *g_w_* := ḡ_K_ d_*_′(V_*_ − *E_K_*), we have

$$\begin{array}{l} \;\frac{dv}{dt}=-g_{v}\;v-g_{w}\;w+I_{0}\\ \tau_{d}{}^{*}\frac{dw}{dt}=v-w. \end{array}$$

To obtain the linearized membrane impedance, we set *I*_0_ = *I_DC_* + *I_AC_* cos(2π*ft*) and solve the above linear equations to yield *V*(*t, f*) = *V_TR_*(*t*) + V_*_ + *V_AC_*(*f*)cos(2π*ft* + η(*f*)), with *V_TR_*(*t*) being the transient response and η(*f*) being the phase lag. The magnitude of the impedance can be calculated as

(9) $$|Z(f)|=V_{AC}(f)/I_{AC}=1/\sqrt{g_{v}^{2}+{\left(2\pi Cf\right)}^{2}+\zeta (f)},$$

where $\zeta (f)=g_{w}\left(\frac{2g_{v}+g_{w}+2C/\tau_{d}^{*}}{1+{\left(2\pi f\tau_{d}^{*}\right)}^{2}}-\frac{2C}{\tau_{d}^{*}}\right)$. For *ḡ_K_* = 0, |*Z*(*f*)| is equal to $1/\sqrt{g_{L}^{2}+{\left(2\pi Cf\right)}^{2}}$, which is the impedance of the simple RC membrane. For large *f*, the membrane impedance |*Z*(*f*)| decays according to 1/2π*Cf* (see Figure 1D).

### AC and noise of the membrane potential

In the preceding sections, we obtained equations for phase-locked synaptic inputs and the effects of the membrane filter. Using these results, we next derive analytical expressions that relate the AC and noise components of the membrane potential to the input parameters, such as the stimulus frequency (locking frequency) *f_s_*, number *M* of presynaptic NM fibers, their mean spike rate λ_0_, their vector strength *r*, the synaptic time constant τ, and the membrane impedance *Z*(*f*).

To calculate the magnitudes of the *AC* (*A_V_*) and noise (*N_V_*) in the membrane potential, we incorporate the linear effects of the driving voltage (*E*_syn_ − V_*_) and the membrane impedance *Z*(*f*). Using Equations 1, 3, 6, and 9, we have

(10) $$A_{V}\text{​}=\text{​}A_{G}|E_{\text{syn}}\text{​}-\text{​}V_{*}\|Z(f_{s})|=\frac{2rD_{G}}{1+{\left(2\pi f_{s}\tau \right)}^{2}}\;|E_{\text{syn}}-V_{*}\|Z(f_{s})|\text{​},\;\;\;\;\;\;\;$$

(11) $$\begin{array}{l} N_{V}=|E_{\text{syn}}-V_{*}|\sqrt{M\lambda_{0}\int_{-\infty }^{\infty }|F_{\alpha }(f){|}^{2}|Z(f){|}^{2}df}\\ \;=\frac{D_{G}|E_{\text{syn}}-V_{*}|}{\sqrt{M\lambda_{0}}}\sqrt{\int_{-\infty }^{\infty }\frac{|Z(f){|}^{2}}{{\left(1+{\left(2\pi f\tau \right)}^{2}\right)}^{2}}df}, \end{array}$$

The holding potential V_*_ here satisfies the equation *g_L_*(*E_L_* − V_*_) + *ḡ_K_d*_∞_(V_*_)(*E_K_* − V_*_) + *D_G_*(*E*_syn_ − V_*_) = 0.

Equations 10 and 11 describe the AC and noise components of the (sound analogue) membrane potential. Both *A_V_* and *N_V_* are linear to the average input *D_G_*. The *AC* amplitude of the membrane potential *A_V_* (i.e., the sound analogue potential) is also linear to that of the synaptic input *A_G_*, because of the linear membrane response. The validity of the linear approximation will be examined in the next section. Although the membrane response is assumed to be linear at each frequency, the noise amplitude of the membrane potential *N_V_* is not linear to that of the synaptic input *N_G_* because the effect of the membrane filter differs between frequencies (i.e., high frequency noise components are more likely to be reduced than low frequency components; see Figure 1D).

### Numerical simulations

In order to test the validity of the theoretical results obtained above, we carried out numerical simulations. The basic settings of our simulation are the same as those in our previous study (Funabiki et al., 2011). Our model consists of NM fibers and an NL cell body, while the phase-locked spiking activity of each NM fiber is modeled as the von Mises distribution. In the following simulations and analyses, we assume that ipsi- and contralateral NM inputs arrive perfectly in-phase. The NL neuron is modeled as a non-excitable single compartment with leak and *K*_LVA_ conductances. The large *K*_LVA_ conductance greatly reduces the membrane impedance in the low frequency region (Figure 1D), yielding a very short membrane time constant of about 0.1 ms. Since the roles of the *K*_LVA_ conductance have been studied and discussed extensively (Manis and Marx, 1991; Reyes et al., 1994; Svirskis et al., 2002; Rothman and Manis, 2003; Day et al., 2008; Gai et al., 2009; Jercog et al., 2010; Mathews et al., 2010), we do not investigate its effects further in this study. The kinetics of the *K*_LVA_ conductance was adopted from a study of the chick NM (Rathouz and Trussell, 1998).

The simulated synaptic input (Figure 3A) is oscillatory and can be decomposed into a signal (AC) component and a noise component. The amplitudes of the DC, AC, and noise components of the simulated synaptic conductance were 21.7, 12.7, and 4.6 nS, respectively. These simulation results agreed well with the theoretical predictions of *D_G_* = 21.7, *A_G_* = 12.7, and *N_G_* = 4.4 nS (Equations 5, 6, and 8). The periodic synaptic input induces an oscillatory membrane potential (Figure 3B). The magnitudes of the AC and noise components of the simulated potential traces were 1.25 and 0.94 mV, respectively. These values matched the theoretical predictions of *A_V_* = 1.25 mV (Equation 10) and *N_V_* = 1.03 mV (Equation 11).

The power spectral densities of the simulated input (Figure 3C) and the membrane potential (Figure 3D) show large peaks at the signal frequency and smaller peaks at higher harmonics. The peak height of the second harmonic of the simulated membrane potential is over two orders of magnitude smaller than the main peak (Figure 3D). The simulated power spectral densities are in excellent agreement with the theoretical prediction (gray curves and filled circles in Figures 3C,D). Due to the low-pass property of the membrane (Figure 1D), noise in the membrane potential consists mainly of the frequency components below the signal frequency. In the accompanying paper (Ashida et al., 2013), we systematically examine the roles of the number of converging NM fiber on the NL neuron, their average spike rate, their degree of phase-locking and the synaptic time constant to investigate how these parameters affect the formation of sound analogue potential in the NL neuron.

### Frequency dependence

**Figure 4:**
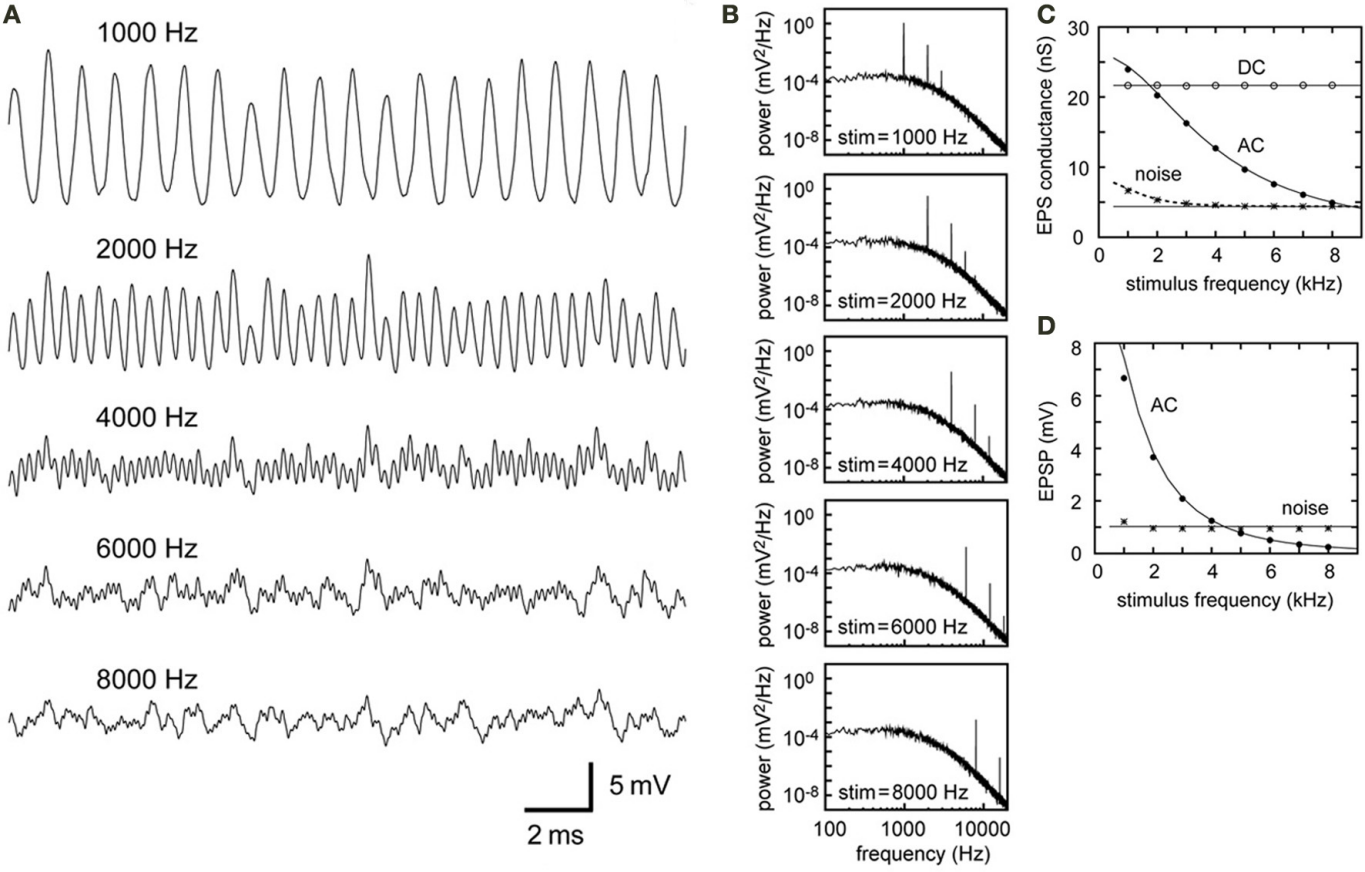
**Simulations of the synaptic input in NL with different stimulus frequencies**. All the parameters except the stimulus frequency are fixed (see Table 2). **(A)** Simulated traces of the model membrane potential. Stimulus frequencies are from 1000 to 8000 Hz, which correspond to the owl's best hearing frequencies. **(B)** PSDs of the five traces shown in **(A)**. Positions and heights of the peaks at the input frequency and higher harmonics depend on the stimulus frequency, while the other frequency components remain unchanged. **(C)** Dependence of the DC, AC, and noise amplitudes of the simulated synaptic input on the stimulus frequency. **(D)** Dependence of the AC and noise components of the simulated membrane potential on the stimulus frequency. The solid lines in **(C,D)** are obtained from analytical calculations without higher harmonics included. The dotted black line in **(C)** is obtained from analytical calculations with the second harmonic included (i.e., $N_{G}^{\prime }=\sqrt{N_{G}^{2}+L_{2}^{2}}$; see Equations 7, 8). The effect of higher harmonics is clear in the noise component of the synaptic input for stimulus frequencies below 2 kHz (shown in **C**), whereas it is not prominent in the membrane potential (shown in **D**).

Simulated sound analogue potentials are frequency dependent (Figure 4A), even when all the other parameters including *VS*, the synaptic time constant and membrane properties are fixed. Effects of these parameters are studied in the accompanying paper (Ashida et al., 2013). For low frequencies (1–2 kHz), AC components are generally large, while for high frequencies (6–8 kHz), the simulated AC amplitudes are less than 1 mV (Figures 4A,D). This dramatic decrease in the AC component is due to the filtering properties of the synapse (Equation 3) and the membrane (Equation 9, Figure 1D). The higher the signal frequency, the more the AC component is diminished by the effect of these low-pass filters (Figure 4B). To obtain a sound analogue potential exceeding 1 mV at over 6 kHz, both the synaptic and membrane time constants must be a few times smaller than the values used in our simulation. Membrane time constants of mammalian outer hair cells decrease with their characteristic frequency (Johnson et al., 2011). Similar frequency dependence may exist in auditory brainstem neurons.

Since all the simulation parameters except frequency are fixed, the baseline noise level of the PSD curve does not change with frequency (Equation 11, Figure 4B). For low frequencies (1–2 kHz), however, the total amount of noise is slightly higher than for other frequencies because of the second harmonic (Figures 4C,D). At the level of synaptic conductance (Figure 4C), the effect of the second harmonic is more prominent than at the level of membrane potential (Figure 4D), where the membrane filter (Figure 1D) further reduces high frequency components. The overall contribution of the second harmonic to the membrane potential noise is therefore limited to frequencies below 2 kHz (Figure 4D). It should also be noted that, for these low frequencies (e.g., Figure 4A, 1 kHz), the simulated traces do not resemble pure sinusoids, because higher harmonics skew the waveform.

Our analytical calculations for the DC conductance (Equation 5), AC conductance (Equation 6) and AC potential (Equation 10) match the simulation results well (Figures 4C,D) for frequencies of 2 kHz and above. For frequencies below 2 kHz, however, there is a slight discrepancy between the theoretical prediction of the membrane AC (7.43 mV) and its simulated value (6.67 mV). Also, for low frequencies, the second harmonic needs to be considered to predict conductance noise precisely (Figure 4C). As mentioned above, the low-pass membrane filter effectively reduces higher harmonics on the membrane potential, resulting in smaller disagreement between the theoretical prediction and the simulation of the noise components (compare the noise amplitudes in Figures 4C,D).

## Discussion

The sound analogue membrane potential, which is created by a “volley” of phase-locked inputs (Wever and Bray, 1930; Joris and Smith, 2008), underlies coincidence detection in the owl's NL neurons (Funabiki et al., 2011). In principle, phase-locked input sequences from the NM axons are filtered by synaptic and membrane processes, inducing oscillatory membrane potentials in NL (Figure 1A). The NL neuron linearly converts the AC signal component of the oscillatory potential into output spike rates (Funabiki et al., 2011). In the present paper, we derive theoretical equations that relate presynaptic, synaptic, and postsynaptic factors with the DC, AC, and noise components of the sound analogue potential, and test the agreement between theoretical predictions and numerical simulations. In the accompanying paper (Ashida et al., 2013), we carry out further simulations and analyses to examine how these factors affect the ITD coding in NL.

### Theoretical formulations

The main aim of this paper is to provide a detailed theoretical description of how phase-locked synaptic inputs lead to oscillatory membrane potentials. Phase-locked spiking activity was modeled as an inhomogeneous Poisson process with a periodic intensity function, and the PSD of the spike sequence was analytically calculated (Equations 1, 2). The presynaptic spikes were summed and then filtered by the synaptic conductance (Equation 3) and the membrane (Equation 9), resulting in the oscillatory membrane potential (Figure 3B). Our model parameters are based on previous results on the owl's auditory system, but the analysis technique used here can be applied to other systems where phase-locking plays a role in information processing. These systems may include the electrosensory lateral line lobe (Kawasaki and Guo, 1996), olfactory system (Stopfer et al., 2003), barrel cortex (Ewert et al., 2008), visual cortex (Gray and Singer, 1989), and the hippocampus (Harris et al., 2002; Diba and Buzsáki, 2008; Mizuseki et al., 2009).

### Agreement of theory and simulation

The power spectral densities of the simulated waveforms also showed excellent agreement with the theoretical predictions, including the peak heights and the overall noise levels (Figures 3C,D). In general, predictions for the membrane potential are worse than those for the synaptic conductance because the effects of the membrane (Equation 9) are further included in the calculation (compare Equations 6, 8 with 10, 11). Especially for sound analogue potentials of over 5 mV (e.g., Figures 4A,D, 1000 Hz), the assumptions for the linear membrane approximation no longer hold, resulting in a discrepancy between the analytical value and simulation results (see Koch, 1999, chapter 10 and references therein for related discussion on the validity of the linear approximation). Thus, the theoretical formulation, which can predict the property of the oscillatory membrane potential without doing computationally-demanding simulations, is most useful when the AC amplitude is within the range of a few mV.

The theoretical predictions for the DC and AC components largely agreed well with the simulation results (Figures 4C,D). The analytical predictions for the noise amplitudes were also comparable to the simulated values but slightly worse than the predictions for DC and AC, because not one but all frequency components contributed to the calculation of the noise amplitude (Equation 11). The prediction performance was also poorer for low frequency AC (2 kHz or below; Figure 4D). These disagreements stem from the fact that the effects of the *K*_LVA_ conductance are most prominent in low frequencies below 2 kHz (Figure 1D). Violating the assumptions of the linear approximation is more likely to affect low frequency parts of the analytical results.

### Frequency dependence

The degree of phase-locking of the presynaptic NM fibers generally decreases with frequency (Köppl, 1997). In order to facilitate comparisons between different frequencies, however, we fixed this parameter to a typical value of 2–4 kHz NM neurons (*VS* = 0.6) in our simulation (Figure 4). Therefore, the amplitude of the AC component in NL neurons from the high best frequency (>6 kHz) regions could be much smaller than a few mV (Figure 4D), implying that high frequency NL neurons should be extremely sensitive to small AC signals. Simulations suggest that axonal Na conductance may amplify high frequency signals (Ashida et al., 2007). However, how and what cellular and synaptic properties of these neurons enable high frequency ITD computation *in vivo* remains to be investigated. In the accompanying paper (Ashida et al., 2013), effects of changing *VS* on ITD coding are examined in more detail.

### Higher harmonics and noise

In our definition, all the higher harmonics are considered noise, because their ITD dependence is different from that of the main AC signal (Ashida et al., 2007; Slee et al., 2010). Previous studies of the chicken NL *in vitro* pointed out that these higher harmonics could hinder ITD coding in NL neurons (Reyes et al., 1996; Slee et al., 2010). Our simulation results suggest that higher harmonics should be considered for frequencies below 2 kHz (Figures 4C,D). For higher frequency NL neurons, the low-pass filter properties of the synaptic and membrane processes effectively cut off the higher harmonics, minimizing their effects on ITD computation. Furthermore, owls' NL neurons with mid-to-high best frequencies (over 3 kHz) recorded *in vivo* do not show a clear second harmonic (Funabiki et al., 2011), suggesting that higher harmonics play little or no role in the owl's computation of ITD at these frequencies. The best frequency of NL neurons in owls ranges up to 7.5–8 kHz (Carr and Konishi, 1990; Peña et al., 2001), whereas the frequency limit of the chicken NL is 3.5–4 kHz (Rubel and Parks, 1975). Thus, the effects of higher harmonics on ITD coding would be more salient in the chicken than in the owl.

The amplitudes of higher harmonics increase non-linearly to the amplitude of the fundamental frequency (Figures 2C,F). For large *VS* values (e.g., *VS* > 0.7), higher harmonics increase more rapidly than the signal, resulting in a faster increase in noise. In our simulation settings, the amplitude of the second harmonic of 1.3 mV at 2 kHz for *VS* = 0.6 increases up to 6.4 mV for *VS* = 1.0, showing a three times faster increase than the AC component at 1 kHz. These results suggest that perfect phase-locking may not always be beneficial to ITD coding. The owl's auditory nerve recordings show a prominent plateau of *VS* about 0.7 at 1.5–3 kHz (Köppl, 1997). This plateau might thus be related to the optimization strategy of the noise level in ITD computation. Noise may affect frequency tuning, temporal coding, and information capacity (e.g., Brunel et al., 2001; Richardson et al., 2003; Butts and Goldman, 2006; Gai et al., 2009; Rossant et al., 2011; See also Faisal et al., 2008; and McDonnell and Abbott, 2009; for recent reviews). Further investigation is necessary to conclude how higher harmonics and other neuronal noise positively or negatively contribute to high frequency ITD detection through oscillatory synaptic inputs.

### Conflict of interest statement

The authors declare that the research was conducted in the absence of any commercial or financial relationships that could be construed as a potential conflict of interest.

## Abbreviations

ITD: interaural time difference
NM: nucleus magnocellularis
NL: nucleus laminaris
VS: vector strength
EPSG: excitatory post synaptic conductance
K_LVA_: low voltage activated potassium.
